# On Tumours of the Uterus and Its Appendages. (Jacksonian Prize Dissertation.)

**Published:** 1847-10

**Authors:** 


*On Tumours of the Uterus and its Appendages.* (*Jacksonian Prize Dissertation.*) By Thomas Stafford Lee, m.r.c.s.e., Fellow of the Medico-Chirurgical Society, &c. &c.—*London*, 1847. 8vo, pp. 262.

Mr. Lee divides the subject of his work into three divisions. Firstly, he considers tumours of the uterus; comprising tumours of the walls of the uterus, polypoid tumours, cauliflower excrescence of the uterus, and malignant tumours of that organ. 2. Tumours of the ovary; comprising cystic tumours of the ovary, and the malignant tumours of that organ. 3. Tumours of the vagina and external organs of generation.

By the term tumour of the uterus, Mr. Lee wishes to describe all those morbid growths which, being at the same time attached to its substance, project into its cavity or from its walls, and divides them into the "benign" and malignant. The former (the non-malignant) he describes also under the term fibrous tumour.

The symptoms of fibrous tumour of the uterus are seldom referable to the viscus itself. There is irritability of the bladder and rectum, the urine cannot be long retained, or there sometimes may be entire retention; the fæces are flattened, and, from a weight in the pelvis, the patient is led to take advice. They are often extremely chronic, and will remain dormant for many years. Virgins are more liable to suffer from them than married women. Of 74 preparations in five museums of the metropolis, examined by Mr. Lee, in 18, or one fourth, the tumour occupied the fundus externally; in another fourth it was situate on the posterior wall; and in another fourth (19 cases) it was found distending the cavity. In four examples, the anterior wall of the uterus was the seat of the morbid growth; in four more the posterior part of the cervix; but in no instance was its anterior portion affected. They are of a cellulo-fibrous structure, and pass into the cartilaginous and osseous transformations. Sometimes the bone is so hard, as to be able to receive a fine polish. When the tumours project into the uterine cavity, and undergo this degeneration, they may become detached and expelled.

Fibrous tumours are insensible, and have but few blood-vessels; it is from the cellular cyst around them that the blood proceeds in those uterine hemorrhages which arise when the tumour protrudes into the cavity of the viscus.

Mr. Lee thus describes the symptoms of fibrous tumours of the uterus:

"Sometimes this disease may exist and only a little leucorrhœa be present, and the morbid product not ascertained until after death; at others, there is a sense of burning heat in the pelvis, running down the thighs, irritability and uneasiness about the rectum; the stomach is disordered, and it is said there is great venereal excitement. The mind becomes affected; there is melancholy, and all the symptoms of hysteria: very frequently there are expulsive pains—and, when present, are considered of great diagnostic value by M. Lisfranc. If you question further, you will find that the functions of the bladder are interfered with; and this perhaps was the first symptom which drew the attention to the womb: a hard tumour can be frequently felt above the pelvis, œdema of the legs is present, and there is a deep-seated weight in the pelvis." (pp. 11-2.)

The symptoms will vary according to the situation of the tumour:

"Those tumours which are placed under the peritoneal surface can be distinctly felt by the hand on external abdominal examination: they may be either smooth or lobulated, and generally, though not always, have a fixed character; but when they possess a pedicle, and it is long, they can be moved about the cavity of the abdomen like a diseased ovarium. If their growth is rapid, they are accompanied with indurations of the uterine tissue around their seat; you then have great pain on pressure, with symptoms of general disturbance, more or less fever, with quick pulse, thirst, and derangement of the digestive system. These symptoms are so frequent when the tumour occupies this position as almost to be pathognomonic of the disease. 2dly. If the tumours are placed in the posterior or lateral parts of the uterus, the early symptoms are more distressing; you have then great pain from pressure on the pelvic nerves, the veins in the abdomen become pressed upon, œdema and varicose veins show themselves, more particularly on one side, sometimes on both; piles are produced; the evacuations of the rectum are interfered with, the fæces are flattened, and constipation, frequently the most obstinate and troublesome symptom, is present, and produces inconveniences we can hardly obviate. 3dly. If the tumours are placed in the anterior walls of the uterus, the functions of the bladder become more particularly implicated: the urine can be retained only for a short time; there is almost constant desire to make water, although only a small quantity is evacuated. At other times retention of urine occurs, which is relieved by change of posture or the catheter. Piles are a very frequent accompaniment of tumours of the uterus. 4thly. If attached to the fundus, it may cause retroversion of the womb (Clarke), and prevent the evacuation of fæces and urine at the same time. Dr. Lever relates a case of this sort, 'where the obstruction had been so great, that the bladder, ureters, and pelvis of the kidneys were enormously distended, while the bowels were loaded with masses of hard, feculent matter.'

"2. When the tumours are placed in the walls of the uterus these symptoms are much more obscure, until they obtain consideration from their bulk. In many instances, the only symptoms referable to the uterus are a weight in the seat of that organ, with occasionally a burning sensation, accompanied by an increased mucous discharge: this may remain for years without producing any further inconvenience, until some cause or other produces irritation; it then increases and produces all the symptoms of pressure on the neighbouring organs, which have been already referred to. When in this position, they may even become osseous without attracting attention.

"3. Tumours projecting into the cavity of the uterus are much more serious in their nature, and produce more immediate effects than those just described. Of these there are two varieties—1st, those which retain their character as tumours, and project into the cavity of the uterus; 2dly, those that lose that character, project so far as to obtain a peduncular attachment to the womb, and in fact become polypoid tumours. These latter, with their symptoms, will be noticed when speaking of polypi.

"When the tumour is submucous, and only projects into the cavity, and not so far as to become a polypoid tumour, the first symptoms usually observed are those connected with derangement of the function of menstruation: the patient finds that the periodical discharge is increased in its quantity, lasts longer than usual, and is followed by a more or less white discharge. This may or may not call attention to the uterus: but soon the symptoms are aggravated, the quantity of discharge is not only increased, but contains clots, a considerable portion of blood is lost at each period, and these occur more frequently, until at last the constitution begins to suffer." (pp. 12-14.)

Not only do hemorrhages take place at the menstrual period, when the tumour projects, but the patient is also harassed by expulsive pains. In addition to these general symptoms, there are those to be ascertained by manual investigation. By this it will be necessary to determine the condition of the os and cervix uteri, the weight, with the mobility or immobility of the uterus, and whether the uterus is connected to any mass previously felt above the pubis. Mr. Lee speaks highly of the uterine sound used by Dr. Simpson, of Edinburgh, in the diagnosis of uterine disease:

"This instrument has the appearance of a small male catheter, with a bulb at one extremity, and fixed in a handle at the other; it is marked by an elevation at $2\frac{1}{2}$ inches from the extremity, that being the natural length of the cavity of the healthy uterus, and is then graduated with inches, in order to ascertain the length of the cavity of a diseased one. When the instrument is passed to its full extent into a healthy uterus, you can elevate the fundus above the pubis, turn it from side to side, and also throw it down upon the rectum; these movements showing the healthiness of the organ: therefore, if they can be made while a tumour exists in the abdomen, it proves that the uterus itself is unconnected with it. In speaking of this instrument, Professor Simpson says, 'But if a tumour is connected to the walls of the uterus, the sound will appear as if it passed into the mass, which will move with all the movements of the instrument, and the uterus itself will be found intimately attached to it; whereas, supposing the tumour unconnected with the uterus, we should be able to perceive that the uterus is moveable and free, and could be thrown down towards the rectum, and easily detached from the tumour.'" (p. 16.)

Mr. Lee points out the necessity and mode of distinguishing fibrous tumours from indurations depending upon an inflammatory state of the uterus, from pregnancy, from ovarian disease, from uterine polypi, and from tumours situate in or upon other viscera of the pelvis or abdomen. Perhaps the most important point in the diagnosis is the distinction between a morbid growth and pregnancy. Mr. Lee observes:

"In all cases of enlargement of the womb, the breasts are the first organs to sympathise with its development, and this is more especially seen in the young and those who have never borne children; so that whether the cause of the distension be the retention of the catamenia or tumour of the uterus, the mammæ become developed and enlarge to a certain extent, as if Nature were providing nutrition for the young. But in pregnancy the nipple and surrounding parts undergo certain changes which are peculiar to that state, and although not infallible, still are very conclusive. These changes consist of an œdematous appearance of the areola and nipple, and an enlargement of the follicles. Some or all of these changes, as far as my experience goes (and I have taken notes of more than one hundred patients), are always present; in two only of the hundred was their neither areola nor follicle, but the œdematous state of the nipple existed to indicate pregnancy. But some of these signs not only belong to pregnancy, but also to disease, and consequently ought to put us on our guard. I have noted carefully the appearances of the breasts in ten cases of fibrous tumours, and I have almost invariably found the following characters. That at the commencement of the disease, or when it had produced such irritation as to cause the patient to apply for medical advice, I have found that the breasts have, in the great majority of cases, been enlarged and tumid; that the areola has in eight cases out of ten been enlarged and darkened; that in the same number the follicles have been more or less numerous, in some they have been remarkably distinct; but that in only one case out of ten was there any moisture or œdema present, and this was a suspicious one, as the patient never came back after the first consultation. All these cases were under observation a considerable time, except the last, and therefore we are positive that in none pregnancy existed. In one case also, the patient obtained by squeezing the nipple a whitish fluid resembling milk.

"The absence of these signs about the nipple are of the greatest importance in our diagnosis; but other points also deserve notice. In pregnancy, the womb has a peculiar elastic, doughy feel, perfectly regular on its surface, and ovoid in its shape. The fœtal heart can be heard beating about the umbilicus, and the placental murmur is usually heard in the left iliac region. But in disease the tumour is irregular, and of stony hardness; no placental murmur nor fœtal heart is heard. The beatings of the aorta may be observed with distinctness, but it can be easily distinguished from the fœtal heart, as it is a single sound, and synchronous with the pulse. In tumours of the uterus the catamenia are present, and usually increased, whilst in pregnancy they are absent; and if any spurious discharge takes place it is scanty." (pp. 20-1.)

*Treatment of fibrous tumours.* In a great majority of cases they are quiescent, and require no notice; when, however, an inflammatory process is set up around the morbid growth, local depletion and the antiphlogistic treatment in general are indicated. Mr. Lee recommends the application of leeches to the neck or body of the womb, and a hip-bath immediately after the leeches come away. He recommends as many as six to be applied once or twice a week.

> "During the intervals of the leechings, mercury or iodine should be applied to the womb itself, either in its pure state or made more consistent with wax. The ointment used at the Red Lion Hospital for Women, is mixed with one part of the Ung. hyd. fort., one part of Cera flava, and one part of lard. This is rolled up in the form of a ball, and introduced into the vagina every night, as high up as possible, in order that it may envelope the os and cervix of the uterus; this remains for twenty-four hours, when it has generally disappeared; it may then be repeated.
>
> "I can strongly recommend this plan of local depletion with the application of mercurial ointment; of course it requires judgment to adapt it to the various cases which come under notice, but in all cases where it is judiciously applied it improves the patient..............Dr. Rigby has carried this plan still further; not only does he envelope the os and cervix uteri with the strong mercury ointment, but he dissolves the ointment, and after having drawn it up into a catheter, injects it into the cavity of the womb. He tells me that this is a great additional advantage, and that he has some cases under his care where, in one case in particular, the tumour which formerly was one large, hard, solid mass, is now separating into distinct parts and becoming gradually less." (pp. 25-6 )

Iodine is, of course, one of the remedies recommended by Mr. Lee, following in the wake of Dr. Ashwell and Dr. Walshe. In very many cases, nothing can be done except to palliate the irritation; if pregnancy take place, the danger is great, and the induction of premature labour becomes necessary, as recommended by Dr. Ashwell.

The second chapter is devoted to the consideration of polypi of the uterus; Mr. Lee discusses them as polypi and polypoid tumours. These do not differ in structure from the preceding class of tumours. The most dangerous symptom connected with them is the profuse hemorrhage to which even small polypi will give rise. Mr. Lee raises the question whence the blood proceeds, and concludes that the bleeding may be attributed to the very vascular state of the mucous membrane at the insertion of the polypus into the uterus; that the veins of the part are the principal sources of bleeding; and when the mucous membrane is absorbed the vascular network which envelops these growths may add materially to the result. Even when the mucous membrane is uninjured, this envelope may materially increase its vascularity.

The soft polypi of the uterus, or the polypi proper, are divided into six species, comprising—1, vesicular polypi; 2, polypi from the enlargement of the nabothian glands; 3, fibro-cellular polypi; 4, cellulo-vascular; 5, mucous polypi; 6, the channelled polypi of the cervix. The symptoms marking these morbid changes closely resemble those which characterise the polypoid tumours, but Mr. Lee remarks, that the white mucous discharges are greater, while the expulsive pains are less, than in polypoid tumours; hemorrhages are very frequent in these polypi, and is the principal symptom to which we ought to direct our attention. Its source is not only from the plexiform arrangement of the veins of the mucous membrane at the base of the tumour, as we find to be the case in the former disease, but also from their own proper vessels, principally veins. The treatment consists in their removal either by excision or ligature, as medicines are useless. In the first instance, however, it will be necessary to repress the profuse hemorrhage to which they give rise, and which indeed will follow excision. Some excellent practical remarks illustrate the chirurgical treatment of polypi.

The cauliflower excrescence of the os uteri comes next under discussion. The following is the result of Mr. Lee's microscopic investigations into its composition in a case which came under his notice:

"Under the microscope these lobules were found to be covered individually by epithelial scales resembling those of the mucous membrane; and each was composed of nucleated cells, with here and there a blood-vessel ramifying on it, but the tumour was not apparently vascular. The edge of the lobules, with epithelial scales, appeared as if impacted one upon another; beneath which, from its circumference, where the cells were much compressed to its centre, cells became gradually developed. There was no appearance of fibrous tissue, nor any of the caudate cells indicating cancer. This then was the result of a careful examination of a part of this tumour removed during life by Dr. Richard Quain and myself. The following is a description of a portion examined in the same way after death: when a piece of the tumour, the only remains of which were in small detached clusters, was taken and placed in water, it appeared to be made up of a number of villi, apparently attached to a central substance of more firm consistence. It was composed of nucleated cells of large size, some circular, some oval, and others elongated oval; these contained a quantity of granular matter and a well-defined nucleus, which appeared to contain a cavity filled with a quantity of granular matter. The two together had the appearance of a cell within a cell, or a compound cell. These cells were connected by fine filaments like cellular filaments. From this examination we conclude that the tumour is composed entirely of cells, and that these are covered by an epithelial membrane; also that it was of simple structure and not malignant." (p. 84.)

It is well known that, in cases of this kind, there has been observed an immense discharge of a watery fluid. Mr. Lee investigated this also:

"It was of a brownish colour, tenacious to the touch, and of a faint odour; it had the appearance, when in large quantities, of saliva coloured. Under the microscope, we found that it was composed of an immense number of nucleated cells, principally of an elongated oval form, containing some granular matter, and each cell was provided with a distinct nucleus; a quantity of granular matter was seen floating in all directions in a thin fluid, which contained a number of epithelial scales. These appearances go far to establish the opinion proposed by Dr. Anderson, that the discharge is dependent on the effusion of cells from the blood-vessels, and thus its great exhausting power is explained." (p. 87.)

Nothing is added by our author to the pathology or treatment of encephaloid, or scirrhous tumours of the uterus.

The second part of Mr. Lee's work treats of diseases of the ovaries; and the first chapter is devoted to ovarian dropsy. In our review of Dr. Clay's pamphlet, (vol. XVI, p. 587) we occupied a considerable space with a consideration of the capital operation of ovariotomy. Four years have brought with them additional experience, and in this chapter of Mr. Lee's we have the whole subject of ovarian disease brought under review, and the treatment by extirpation of the sac, as well as by other means, fully investigated.

Mr. Lee collected 140 cases of ovarian dropsy, and placed their characteristics in a tabular form; some he observed for himself, the details of others are from books.

As regards the effect of marriage, it appears that of 136 examples, 88 were married, and 11 were widows, so that only 27·2 per cent. were single. As regards age, only 3 were those of females under twenty years; but between twenty and forty, there were 82 examples, 26 between forty and fifty, and 19 between fifty and sixty. Mr. Lee has omitted to state what ratio these numbers bear to the number of females living at the respective ages, an important point in estimating the *influence* of age.

With regard to other causes, Mr. Lee deduces from a rather meagre table, that the most frequent is parturition, that the sudden suppression of the menses is next in frequency, the excitement of marriage follows as a third, and he believes that disappointed affection is one of the most fertile causes in those that are unmarried.

The right ovary is more usually affected than the left, for of 93 cases, while only in 8, or 8·6 per cent. both ovaries were diseased, in 50, or 58·8 per cent. of the remainder, the right was affected exclusively.

Mr. Lee considers the pathology of ovarian dropsy under the following heads: 1. The simple cyst attached to the ovary or broad ligaments of the uterus. 2. Enlargement of the graafian vesicles. 3. Cysts unconnected with the ovary, and which are formed in various parts of the abdomen, usually mistaken for ovarian dropsy. And lastly, 4, multilocular cysts. We shall not follow him through the pathological anatomy of these various structures, nor through his consideration of their contents, and their diagnosis, but pass at once to their treatment.

In the early stages, when the enlargement of the ovary arises from inflammatory action, the enlarged organ can be felt distinctly between the vagina and rectum, as a tumour very painful on pressure. Under these circumstances, the antiphlogistic treatment is indicated:

"Local bloodletting is very important; this is to be effected, not as it is usually prescribed, viz., by cupping the loins, or by application of leeches to the vulva, but by the actual application of leeches to the tumour itself through the rectum. The bowel is to be washed out by a copious enema; then, by placing the leeches in a long tube, the upper end of which is perforated by a number of holes, the mucous membrane bulges through these perforations on introducing it into the bowel, and the leeches usually fix themselves to it, as is necessary in introducing this instrument; and when it arrives at the diseased ovary it produces pain, and ought not to be pushed further. Large quantities of blood may be taken in this way, and the application ought to be repeated every fourth day. This local depletion is to be further assisted by calomel, and opium, and salines Blisters and leeches ought to be applied to the tumour, when above the pubis, when pain and uneasiness are felt." (p. 155.)

When the tumour has so far increased as to occupy the cavity of the abdomen, the antiphlogistic treatment may still be useful, combined with counter-irritation in various ways. Iodine is of course mentioned as a powerful remedy, and its beneficial influence seems to be due to the suppuration of the cyst which follows its use. This result cannot be attained, however, without dangerous constitutional disturbance. Moderate bandaging, &c., is not estimated highly by Mr. Lee; and it appears, from his inquiries, that some of the cases published by Mr. Isaac B. Brown as being successfully treated by his method (one principal point of which is the careful application of a tight flannel bandage) were not so successful as his statements would imply; that, in fact, his conclusions were somewhat premature.

The operation of tapping is fully considered by Mr. Lee, and is shown statistically to be an operation much more dangerous than is generally supposed. After mentioning minor causes of this, Mr. Lee observes:

"The greatest danger to be apprehended after tapping, is the inflammation of the cyst itself, or sometimes of the peritoneum of the abdomen. This is almost the inevitable termination, some time or other, of the lives of the patients who are subjected to tapping; according to my experience, the cyst itself is the part most usually inflamed. In some cases a portion of the ovarian fluid escapes, and acts as a foreign body on the peritoneum; in others, the internal lining of the cyst takes upon itself an inflammatory action, or the trocar may have punctured a mass of cysts, and thus produced inflammation. The effects of the inflammation, however produced, are alarming; all the symptoms of active fever are found; there is great pain in the abdomen, which becomes tense and very tympanitic, vomiting ensues, and rapid exhaustion takes place, followed by death."

Mr. Lee has collected a number of cases where the duration of the disease, after the first tapping, was accurately recorded, and finds that more than one half of those who died did so within four months, and nearly half of these were only tapped once: almost all the deaths after the first operation were attributed to the inflammation of the sac or peritoneum.

Combining the results of his own tabulation and of Mr. Southam's, it would appear that the mortality after tapping is much greater than is generally believed: 24 out of 57 deaths died after the *first* tapping, and within eight months after the operation; 20 of the 24 died within one month, and 12 of these 20 within one week. From these and other observations, Mr. Lee concludes that, taken at its best, tapping is a very dangerous means of palliating ovarian dropsy; that when it is had recourse to, it will have to be frequently repeated; that the relief afforded between each operation will become gradually less, and the dangers consequently greater. The operation ought only to be performed under one of two circumstances: either early, when the cyst is unilocular, or when the ovarian tumour is producing fatal pressure upon vital organs. In no case, except under the latter circumstances, ought a multilocular cyst to be punctured, because the relief given is so trifling, and the dangers of tapping are so much increased in this form of the disease.

The radical cure by excision of the sac is elaborately considered by Mr. Lee. Previously to ascertaining the results of the operation, he undertakes the task of determining the usual course of the ovarian disease, and the results of the ordinary treatment. The dangers of the operation of tapping have been stated already. Under the ordinary treatment, the duration of the disease was found to be as follows: 26 per cent. lived one year; 19 per cent. two years; 13 per cent. three years; 8 per cent. four years; 2·3 per cent. five years. In fact, half the cases terminate by death in two years. Mr. Lee has tabulated 118 cases treated by excision of the sac (in the Appendix a valuable and elaborate table is given of all the known operations from 1809 to 1846, comprising the leading points in each case), and discusses the various details of the operation with great acumen and judgment. We subjoin his general conclusions, premising that of 114 operations 40 died, being a mortality of 1 in 3.

"That of these 114 operations, in 24, or rather less than one in five, the operation was obliged to be abandoned, either from extent of adhesions, from the tumour being an uterine or omental one, or from there being no tumour at all; proving most indisputably the difficulties of the diagnosis.

"That in the 90 cases where the tumour was removed, nearly one died to three recoveries.

"That the diagnosis of ovarian tumours is very obscure as regards adhesions, and the character of the tumour; that adhesions existed in 46 of the 81 where the fact is mentioned, and in 6 there was no tumour.

"That where adhesions existed, the mortality was greater, being one death in $2\frac{1}{4}$, whereas, the mortality was one in three where they were absent.

"That the disease may be complicated with organic disease of other viscera.

"That the principal recorded causes of death, where it took place soon after the operation, are hemorrhage and peritonitis; but the cases are much too few to be depended upon.

"When death takes place in consequence of the operation, it is very rapid. Of 30 patients, where the time is mentioned, 14 died within 36 hours, and 25 within a week.

"That the character of the disease is of importance with regard to its mortality. In the extraction of hard tumours of the ovary, the mortality was more than 1 in 2. Of the 16, 9 were cured, 7 died, and in 5 the tumour was not removed. Whereas, where the tumour was composed partly of fluid and partly of solid matter, viz. in 65 cases, 44 were cured, 21 died, and in 14 the tumour was not extracted, making the mortality less than one in three: so that encysted dropsy is much more favorable to the operation than hard tumours of that organ." (p. 210.)

Mr. Lee thinks that, in the majority of cases, the operation of ovariotomy is "decidedly unjustifiable;" but that in others it "is *very* justifiable," namely, in those cases of encysted tumour which have enlarged to such an extent as to demand active interference, or when an unilocular cyst is becoming multilocular. In such examples, with a correct diagnosis, general good health, and freedom of the tumour from adhesions, the operation ought to be performed.

Malignant tumour of the ovary, tumours of the vagina and of the external organs of generation are then considered. The latter class of organs can scarcely be considered as "appendages to the uterus," and are only admissible as rendering the work a complete monograph on tumours of the sexual organs of the female. As such we estimate it; and we think it deserves a place in the library of the practitioner, both as a practical treatise and as a work of reference. It is in truth as valuable for the results of thoughtful reading contained in its pages, as for new researches. We have often thought that a very complete series of monographs might be written, if the results of observation and treatment, scattered through our vast periodical literature in the form of "cases," were classified so as to afford general deductions. We ought to add, that original "cases" illustrate several of the diseases treated of in the volume before us.

